# Nitric Oxide and Reactive Oxygen Species in the Pathogenesis of Preeclampsia

**DOI:** 10.3390/ijms16034600

**Published:** 2015-03-02

**Authors:** Keiichi Matsubara, Takashi Higaki, Yuko Matsubara, Akihiro Nawa

**Affiliations:** 1 Department of Obstetrics and Gynecology, Ehime University School of Medicine, Ehime 791-0295, Japan; E-Mails: takeyu@m.ehime-u.ac.jp (Y.M.); nawa2011@m.ehime-u.ac.jp (A.N.); 2 Department of Pediatrics, Ehime University School of Medicine, Ehime 791-0295, Japan; E-Mail: higaki@m.ehime-u.ac.jp

**Keywords:** endothelium, inflammation, nitric oxide, preeclampsia, reactive oxygen species

## Abstract

Preeclampsia (PE) is characterized by disturbed extravillous trophoblast migration toward uterine spiral arteries leading to increased uteroplacental vascular resistance and by vascular dysfunction resulting in reduced systemic vasodilatory properties. Its pathogenesis is mediated by an altered bioavailability of nitric oxide (NO) and tissue damage caused by increased levels of reactive oxygen species (ROS). Furthermore, superoxide (O_2_^−^) rapidly inactivates NO and forms peroxynitrite (ONOO^−^). It is known that ONOO^−^ accumulates in the placental tissues and injures the placental function in PE. In addition, ROS could stimulate platelet adhesion and aggregation leading to intravascular coagulopathy. ROS-induced coagulopathy causes placental infarction and impairs the uteroplacental blood flow in PE. The disorders could lead to the reduction of oxygen and nutrients required for normal fetal development resulting in fetal growth restriction. On the other hand, several antioxidants scavenge ROS and protect tissues against oxidative damage. Placental antioxidants including catalase, superoxide dismutase (SOD), and glutathione peroxidase (GPx) protect the vasculature from ROS and maintain the vascular function. However, placental ischemia in PE decreases the antioxidant activity resulting in further elevated oxidative stress, which leads to the appearance of the pathological conditions of PE including hypertension and proteinuria. Oxidative stress is defined as an imbalance between ROS and antioxidant activity. This review provides new insights about roles of oxidative stress in the pathophysiology of PE.

## 1. Introduction

**Figure 1:**
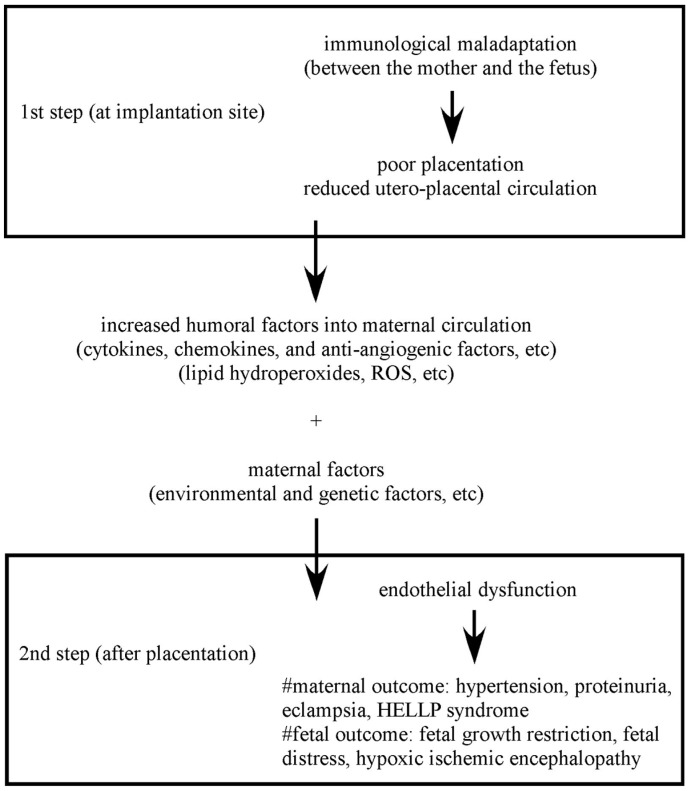
Two-step theory in the pathogenesis of preeclampsia (PE). Immunological maladaptation causes poor placentation leading to placental hypoxia at the first step. Hypoxic placenta induces the production and secretion of humoral factors into maternal systemic circulation. These factors disturb vascular endothelial function resulting in multiple organ failure at the second step. (This figure is modified from [5]).

Preeclampsia (PE) is a pregnancy-induced hypertensive disorder associated with proteinuria that occurs after the 20th gestational week. It affects 7%–10% of pregnancies, and this major disorder is highly associated with perinatal morbidity and mortality [1,2]. Although the etiology of PE has not been entirely clarified, vascular dysfunction resulting in poor placentation, is thought to be its main cause [3,4]. It has been proposed that PE is a two-stage disorder (Figure 1) [5]. The process of PE pathogenesis appears to begin with a malfunction during the trophoblast invasion, followed by disordered neovasucularization. Subsequently, vascular dysfunction of the placenta results in placental secretion of humoral factors into the maternal systemic circulation. These humoral factors trigger multiple organ injuries responsible for the clinical manifestations of PE.

Oxidative stress has been proposed as one of the key humoral factors generated by the poorly perfused placenta [6]. Oxidative stress can induce the adhesion of leukocytes and platelets to the endothelium as well as the release of cytokines and anti-angiogenic factors. Adhesion of blood cells and endothelial cells is critical in the inflammation process involved in the pathogenesis of PE. As a result of the inflammation, generalized vasoconstriction and increased resistance in the placental circulation can be caused by reduced uteroplacental blood flow followed by placental dysfunction [7]. In general, the impairment of circulatory homeostasis in PE is caused mainly by vascular endothelial dysfunction [3], characterized by vasoconstriction that is triggered easily, and low anticoagulant activity. Reactive oxygen species (ROS) seem to play a critical role in the endothelial dysfunction associated with PE.

In normal pregnancy, nitric oxide (NO) contributes to the maintenance of vascular tone to increase uterine blood flow [8]. Endothelium-dependent vasodilatation is mediated in part by NO and is up-regulated during pregnancy because of the increased estrogen level [8,9]. Endothelial cells produce NO, which is a strong vasorelaxant and anticoagulant factor. Its effect is mediated by guanosine 3',5'-cyclic monophosphate (cGMP) produced by soluble guanylyl cyclase. cGMP activates protein kinase A (PKA) and protein kinase G (PKG) [10,11]. Activated PKA and PKG induce smooth muscle relaxation through the attenuation of myosin light chain kinase activity and augmentation of myosin light chain phosphatase activity, which induces dephosphorylation of the 20-kDa, regulatory, myosin light chain [10,11].

The activation of NO synthase (NOS) triggers the production of NO during pregnancy. NOS consists of three isoforms, including neuronal NOS (nNOS), endothelial NOS (eNOS), and inducible NOS (iNOS). The eNOS isoform is expressed constitutively in the vascular endothelium and maintains vascular tone through the intrinsic synthesis of NO, thereby inhibiting the adhesion of leukocytes and platelets to the endothelium impeding the proinflammatory state. In contrast, iNOS is stimulated in a proinflammatory or an inflammatory condition and produces a temporary excess of NO. Endothelium-derived NO dysfunction has been implicated as a potential cause of PE [12].

Vascular function is modulated by the interference of ROS and NO. Increased ROS production seems to suppress the expression and function of eNOS [13]. Further, peroxynitrite (ONOO^−^) is formed as result of NO scavenging by ROS. The resulting ONOO^−^ not only oxidizes DNA, proteins, and lipids but also interferes with important vascular signaling pathways. Thus ROS increase and subsequent increased ONOO^−^ formation are known to reduce the bioavailability of NO and cause endothelial dysfunction. These effects could be key elements in the pathogenesis of PE.

The interaction between NO and ROS modulates vascular tone. Thus the altered balance of NO and ROS also seem to play a critical role in the pathogenesis of PE [14]. These presumptions suggest that PE can be resolved by removal of the placenta because this organ is a major source of NO and ROS.

## 2. Oxidative Stress in the Pathogenesis of Preeclampsia (PE)

Preeclamptic placenta is known to be hypoxic and stimulate the release of a large amount of syncytiotrophoblast microparticle (STBM) [15]. Placental hypoxia and STBMs could stimulate the production of damage-associated molecular pattern (DAMP), which activates immunocytes including neutrophils and dendritic cells. Activated immunocytes could produce proinflammatory cytokines including tumor necrosis factor α (TNFα) and promote oxidative stress through neutrophil nicotinamide adenine dinucleotide phosphate (NADPH) oxidase activation. Recently, it has also been reported that increased advanced glycation end products (AGEs) interacts with the receptors (RAGE) in PE and activates NADPH oxidase [16]. RAGE/NADPH oxidase-dependent pathway promotes sFlt-1 expression in trophoblasts and is involved in the increased oxidative stress in PE placenta.

### 2.1. Reactive Oxygen Species (ROS)-Producing Enzymes in PE Pathogenesis

Neutrophils in peripheral circulation of patients with PE are known to be activated [17,18]. Activated neutrophils produce ROS through the action of several activated enzymes, including NADPH oxidase [19,20], xanthine oxidase (XO), and uncoupled eNOS [21].

#### 2.1.1. NADPH Oxidase

It is reported that NADPH oxidase in lymphoblasts isolated from PE patients is much more sensitive on agonist stimulation with PMA than in lymphoblasts from normal pregnant women [22]. NADPH oxidase catalyzes the production of ROS from oxygen and NADPH in neutrophils and endothelial cells [23]. ROS produced by NADPH oxidase in moderate concentrations act as signaling molecules to regulate vascular tone [24]. However, excessive ROS production leads to oxidative stress and vascular dysfunction [25]. The integral membrane component of NADPH oxidase is composed of a large subunit, gp^91phox^ (glycoprotein91–phagocyte oxidase), and a small subunit, gp^22phox^. We demonstrated that sera derived from patients with PE stimulated the expression of gp^91phox^ of vascular NADPH oxidase via TNFα and angiotensin II receptor subtype 1 (AT1) [23,26]. It is thought that neutrophil and vascular NADPH oxidase is a key contributor to oxidative stress induced by several inflammatory substances in PE pathogenesis.

#### 2.1.2. Uncoupled Endothelial Nitric Oxide Synthase (eNOS)

The eNOS-induced NO production regulates blood pressure and decreases leukocyte-endothelium adhesion chronically as the first step of inflammation [27]. However, uncoupled eNOS could generate ROS rather than NO [21]. l-arginine is a substrate that NOS utilizes for tetrahydrobiopterin (BH4) binding to the oxygenase domain of eNOS. Then, BH4 stabilizes the dimer as one of the cofactors of NOS activation [28]. In the presence of sufficient BH4, abundant NO can be produced by the eNOS dimer, leading to ROS starvation. However, in the absence of BH4, eNOS dimer would be uncoupled into two monomers. Uncoupled eNOS is less efficient in the production of NO and generates large amounts of ROS. BH4 is thought to maintain the balance between NO and ROS production in the vascular endothelium [29] and the imbalance leads to hypertension. Moreover, it is known that increased asymmetric dimethyl-l-arginine (ADMA) is observed in endothelial cells derived from patients with hypercholesterolemia [30]. ADMA can inhibit eNOS activity through uncoupling eNOS and reduce l-arginine uptake into endothelial cells. Both changes reduce eNOS-induced NO generation. Furthermore, increased plasmatic ADMA is associated with oxidative stress increase and endothelial dysfunction [31]. This uncoupling might play a critical role in the pathogenesis of cardiovascular diseases such as hypertension [21], as well as in the pathogenesis of PE by endothelial dysfunction [32].

#### 2.1.3. Xanthine Oxidase (XO)

It is reported that ischemia/reperfusion markedly stimulates endothelial XO activity leading to increased production of superoxide and peroxide [33,34], which induces cellular damage, immune activation, and vascular dysfunction, such as altering endothelium-dependent vascular relaxation [35,36]. Endothelial XO activity is modulated by xanthine dehydrogenase through the conversion of xanthine dehydrogenase to XO [37]. Furthermore, the increased activity of XO has been observed in the cytotrophoblasts derived from placentas of patients with PE [38]. Endothelium and cytotrophoblasts are important sources of ROS, generated by XO in the pathogenesis of PE.

### 2.2. ROS in the Pathogenesis of PE

Although the ischemic placenta in PE is known to induce ROS production [39,40], ROS is also produced in the systemic vasculature at the second stage of PE pathogenesis [41]. In some studies, placental homogenates derived from patients with PE showed 39% higher hydrogen peroxide production than those derived from normal pregnant women [42]. Additionally, increased ROS concentrations in patients with PE have been proven by the increased levels of malondialdehyde, an index of lipid peroxidation [43]. Lipid peroxidation is closely related to heat-shock protein 70 (HSP70). HSP70 level in the peripheral blood is significantly higher in both fetal and maternal circulations in PE [44]. ROS promotes lipid oxidation and induces the expression of stress proteins such as HSP70. Subsequently, HSP70 might act as the secondary line of defense in systems with compromised antioxidant function. Furthermore, we demonstrated that the level of creatol, a hydroxyl radical adduct of creatinine, began to increase from the first trimester through the third trimester [23]. These results show that oxidative stress is increased in early pregnancy even before the onset of PE. In the vasculature of patients with PE, ROS production is also modulated by AT1 and TNFα [23]. Since AT1 activation and TNFα secretion are stimulated in PE [23,45,46], ROS must be produced significantly in the endothelium and trophoblasts of patients with PE.

## 3. NO during Normal Pregnancy and in the Pathogenesis of PE

The catalysis of the guanidino nitrogen atom of l-arginine by NOS results in NO production and activation of guanylate cyclase, with the subsequent increase of intracellular cyclic guanosine monophosphate (cGMP). cGMP induces smooth muscle cell relaxation and endothelium-dependent vasodilatation [47]. This particle is also responsible for the anti-aggregation of platelets [48] and anti-inflammation [49]. In the endothelium, NO is constitutively produced under physiological conditions through eNOS activation, which is Ca^2+^-dependent. NO produced by eNOS might be ordinarily beneficial in the peripheral circulation and prevent tissue damage [50]; However, inflammatory cytokines inhibit eNOS, causing vasoconstriction in the peripheral circulation [51]. Calcium-independent iNOS plays a pivotal role in inflammation and is usually not expressed in the endothelium. However, inflammation induces the expression of iNOS in the vascular endothelium resulting in excessive NO production [52] through increased inflammatory cytokines such as interleukin-1 (IL-1), IL-6, TNFα, and interferon γ (INFγ) [53]. Additionally, the increased NO derived from iNOS plays a major role in organ rejection, such as that observed in cases of cardiac transplant [54]. The iNOS isoform also plays a predominant role in the leukostasis resulting in vascular dysfunction through ICAM-1 upregulation [55]. Such NO excess might contribute to the multiple organ failure in PE through acceleration of inflammation and other immune reactions.

The participation of NO in the regulation of renal and glomerular hemodynamics [56] has also been described. In fact, NO is an important physiological mediator of the renin-angiotensin system via AT subtype 2 (AT2), which is counter-regulatory to the actions mediated by AT1. Activated AT2 triggers a vasodilator cascade, including the NO/cGMP pathway [57], via increase in NOS mRNA and protein leading to NO production. Conversely, the signaling pathway of AT1 negatively regulates NO production in the endothelium and causes vasoconstriction [58]. In addition, inhibition of NO synthesis, such as that caused by an l-arginine antagonist, stimulates vasoconstriction and results in hypertension [59].

### 3.1. Role of NO in Physiological Condition during Normal Pregnancy and in the Pathogenesis of PE

It is reported that eNOS and iNOS were expressed mainly on syncytiotrophoblasts and endothelial cells in the placenta during pregnancy [60,61]. During normal pregnancy, increased estrogen stimulates endothelium-dependent vasodilatation, mediated in part by NO, which was produced and secreted by activated eNOS [9,62]. eNOS-derived NO contributes to the maintenance of circulatory homeostasis through vascular smooth muscle relaxation for increased uterine blood flow [8] and uterine myometrial quiescence [63]. Blood pressure in normal pregnant women is slightly reduced in the middle of pregnancy in part by increased flow-mediated dilatation (FMD). FMD caused by shear stress stimulates vascular eNOS activation and temporally increased NO production; thus, vessels can be dilated easily during normal pregnancy [64].

Vascular eNOS-derived NO can be a vascular protective agent against inflammation by decreasing the expression of adhesion molecules, including ICAM-1, vascular cell adhesion molecule-1, E-selectin, and P-selectin [65,66,67]. In PE, these adhesion molecules that accelerate inflammation are strongly expressed [45] and inflammation is induced in systemic vasculature and placenta causing uteroplacental perfusion failure [68].

Serum NO concentration is reduced in patients with PE during the first trimester [23]. In early pregnancy, it is thought that vascular dilatation and growth is disturbed leading to poor placentation because of low levels of NO. However, we observed that the serum level of NO in normal pregnant women was similar to that in patients with PE during the third trimester after the onset of PE through vascular iNOS activation [23]. By contrast, the eNOS-derived vascular dilatation was reduced in patients with PE since FMD was disrupted [23]. This discrepancy is explained by the reduced bioavailability of NO. For example, when an excess of both NO and ROS is produced, ROS scavenge NO rapidly and form ONOO^−^ anions and an ultimately decreased vascular NO availability [69].

Although the interaction between NO and ROS could regulate physiological vascular tone during normal pregnancy [70], it is possible that the imbalance between NO and ROS is involved in the pathogenesis of PE.

## 4. ONOO^−^ in PE

Rapid scavenging of NO by ROS leads to the formation of ONOO^−^. The formation of ONOO^−^ in peripheral circulation seems to be involved in hypertension [71]. High levels of ONOO^−^ oxidize and damage DNA, proteins, and lipids, whereas, low levels of ONOO^−^ interferes with vascular signaling pathways involving NO, prostaglandins, calcium ion, MAP kinase, and NF-κB [72]. Further, ONOO^−^ induces eNOS uncoupling by oxidation of BH4 to BH3 and compromises eNOS activity [21]. Furthermore, ONOO^−^ can lead to irreversible nitration of tyrosine residues on other proteins, causing impaired phosphorylation and enzymatic dysfunction.

Not only does ONOO^−^ formation inhibit NO bioavailability but also prostaglandin I2 (PGI2) production through the tyrosine nitration and the prostaglandin synthase (PGIS) inhibition [73] that causes smooth muscle contraction and activation of platelets and white blood cells [74]. Thereafter, the aortic expression of NADPH oxidase and iNOS were markedly increased, paralleled by increases in superoxide and extensive NO in the aorta [75]. Such a combination of decreased NO availability and endothelium-derived superoxide increase induces vascular dysfunction and vasoconstriction [76]. We showed that nitrotyrosine was expressed strongly in endothelial cells and trophoblasts of placenta derived from patients with PE. These results indicate that NO bioavailability is disrupted by ROS and the metabolite ONOO^−^ impairs vascular function and growth associated with poor placentation in PE.

## 5. Antioxidant System

It is known that placental oxidative stress is increased in PE as early as 8 to 10 weeks of gestation. However, endogenous antioxidant activities like glutathione peroxidase (GPx), superoxide dismutase (SOD), and catalase increase in normal early pregnancy [77]. It has been reported that the activity of antioxidants including vitamin E, vitamin C, glutathione (GSH) [43], superoxide dismutase (SOD), and thioredoxin (Trx) was disrupted in PE. Glucose-6-phosphate dehydrogenase, GPx, and glutathione *S*-transferase were also reported as important antioxidant enzymes in PE.

### 5.1. Vitamins (Vitamin C, E)

Vitamin C (ascorbate) is an essential nutrient that acts as an antioxidant, and contributes to the protection of organs against oxidative damage. Although the level of plasma ascorbate is already decreased in normal pregnancy, the level is decreased further in PE [78] and also the level in placental tissue derived from patients with PE is decreased [79]. On the other hand, ascorbate is easily oxidized to ascorbyl radical and then to dehydroascorbate, which generates oxidative stress leading to tissue injury [80]. Oxidized ascorbate is increased in PE plasma and might contribute to vascular dysfunction [78].

Vitamin E is known to reduce ONOO^−^ through the supply of an electron [81]. Serum concentration of vitamin E and the level in placental tissue are decreased in severe PE, meaning there is a decreased antioxidant activity in this condition [82].

### 5.2. Superoxide Dismutase (Mn-SOD, CuZn-SOD, EC-SOD)

SOD is an enzyme that catalyzes the dismutation of the superoxide radical into oxygen or hydrogen peroxide (2O_2_^−^ + 2H^+^ → O_2_ + H_2_O_2_). Mn-SOD is constitutively expressed in the mitochondria and scavenges superoxide radicals, and CuZn-SOD is located in cytoplasm and secreted into the extracellular space. Extracellular SOD (EC-SOD) is synthesized by only a few cell types including vascular smooth muscle cells and is localized in the extracellular matrix of various tissues including the vascular wall and placenta [83]. SOD can react with NO and ROS as an antioxidant; however, as a result, it generates strong ONOO^−^ because NO can react with superoxide three times faster than with SOD [84]. SOD is increased during normal pregnancy [77], and, SOD activity and mRNA expression of CuZn-SOD in the placental tissue derived from patients with PE is decreased, which may result in increased oxidative stress in the placenta of patients with PE [85]. EC-SOD plays a critical role in modulating vascular function in the resistance vessels. It is reported that the mutant allele carriers of the EC-SOD Ala40Thr SNP have an increased risk of severe fetal growth restriction-complicated PE [83]. It is also known that SOD is decreased in erythrocytes derived from patients with PE [86].

### 5.3. Catalase

Catalase degrades hydrogen peroxide into water and oxygen (2H_2_O_2_ → 2H_2_O + O_2_) and thus, reduces the level of ROS. A low concentration of hydrogen peroxide acts as a cellular messenger in insulin signaling pathways, whereas a high concentration is toxic to the pancreatic cells [87]. Furthermore, catalase is an important enzyme of hydrogen peroxide degradation in erythrocytes, and its deficiency causes increased hydrogen peroxide production in many organs, such as in type 2 of diabetes mellitus [87].

During pregnancy, catalase activity is correlated with gestational age; However, the activity plateaus at approximately 12 weeks of gestation [77]. On the other hand, the catalase activity in erythrocytes is decreased in PE [88], which is critical to metabolize hydrogen peroxide in systemic circulation.

### 5.4. Glutathione Peroxidase (GPx)

GPx is known to reduce lipid hydroperoxide and hydrogen peroxide (2Glutathione + H_2_O_2_ → Glutathione disulfide + 2H_2_O) and contributes to organ protection against oxidative stress. Eight isoforms have been identified and GPx1 is highly expressed in many organs.

GPx activity is increased in the placental tissue derived from normal pregnant women [77], and the activity and mRNA is decreased in that derived from patients with PE (GPx1, GPx3, GPx4) [89]. The data of erythrocyte GPx is controversial. Haque *et al.* reported that erythrocyte GPx was reduced in patients with PE [90]. Conversely, Diedrich *et al.* reported that it was increased in patients with HELLP (hemolysis, elevated liver enzymes, and low platelet count) syndrome [91]. Still, GPx deficiency is thought to be involved in the pathophysiology of PE, because its decreased activity is associated with the synthesis of lipid peroxides and thromboxanes, which are increased in PE placenta [92].

## 6. Conclusions

**Figure 2:**
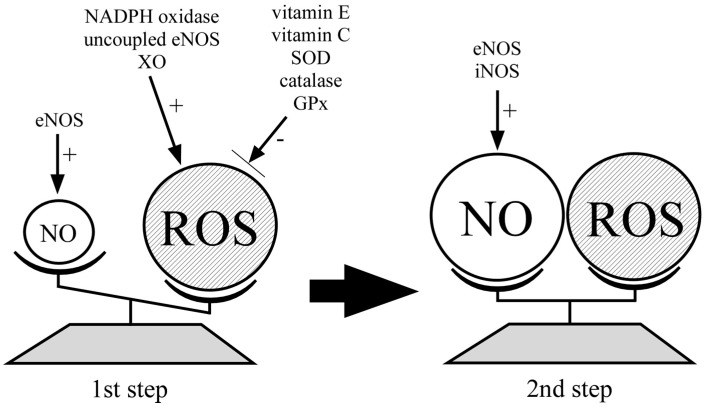
The balance between NO and ROS in the pathogenesis of PE. ROS production is increased and NO production is decreased in the peripheral blood and placenta derived from women who subsequently develop PE at the first step. Both ROS and NO production are increased in women with PE at the second step.

Oxidative stress is a result of a large amount of ROS exceeding antioxidant activity and one of the risk factors for the development of PE through vascular dysfunction. Although poor invasion of cytotrophoblast into uterine myometrium and disturbed spiral artery remodeling plays an important role in the pathophysiology of PE resulting in placental hypoxia, massive quantities of ROS and reduced bioavailability of NO via local RAS [93] and TNFα [94] is involved to the placental hypoxia in early pregnancy and the development of clinical manifestations in women with PE [23] (Figure 2). Elevated levels of ROS can produce protein tyrosine nitration after the onset of PE and iNOS-derived NO might be combined with ROS to generate ONOO^−^ in women suffering from PE. The production of cytotoxic ONOO^−^ could be a hallmark of vascular injury in PE. These findings suggest that the imbalance of ROS and NO results in vasodilatory dysfunction in PE. At this time, the termination of pregnancy is the only way to avoid maternal eclampsia and fetal distress in PE. However, some kind of supplementation such as antioxidants may prevent the occurrence of PE or repair the manifestation of PE in future. In order to establish the role of ROS and NO in the pathogenesis of PE and to evaluate the efficacy of antioxidants on PE, more research is needed.

## Abbreviations

NO: Nitric oxide
ROS: Reactive oxygen species
PE: Preeclampsia
TNFα: Tumor necrosis factor α
cGMP: Guanosine 3',5'-cyclic monophosphate
PKA: Protein kinase A
PKG: Protein kinase G
NOS: Nitric oxide synthase
nNOS: Neuronal NOS
eNOS: Endothelial NOS
iNOS: Inducible NOS
ONOO^−^: Peroxynitrite
ICAM-1: Intercellular adhesion molecule 1
LFA-1: Lymphocyte function-associated antigen 1
MAC-1: Macrophage-1 antigen
NADPH: Nicotinamide adenine dinucleotide phosphate
XO: Xanthine oxidase
AT1: Angiotensin II receptor subtype 1
AT2: Angiotensin II receptor subtype 2
FMD: Flow-mediated dilation
BH4: Tetrahydrobiopterin
ADMA: Asymmetric dimethyl-l-arginine
IFNγ: Interferon γ
PGI2: Prostaglandin I2
GPx: Glutathione peroxidase
GSH: Glutathione
SOD: Superoxide dismutase
Trx: Thioredoxin
Mito-T (4): Mito-Tempol
